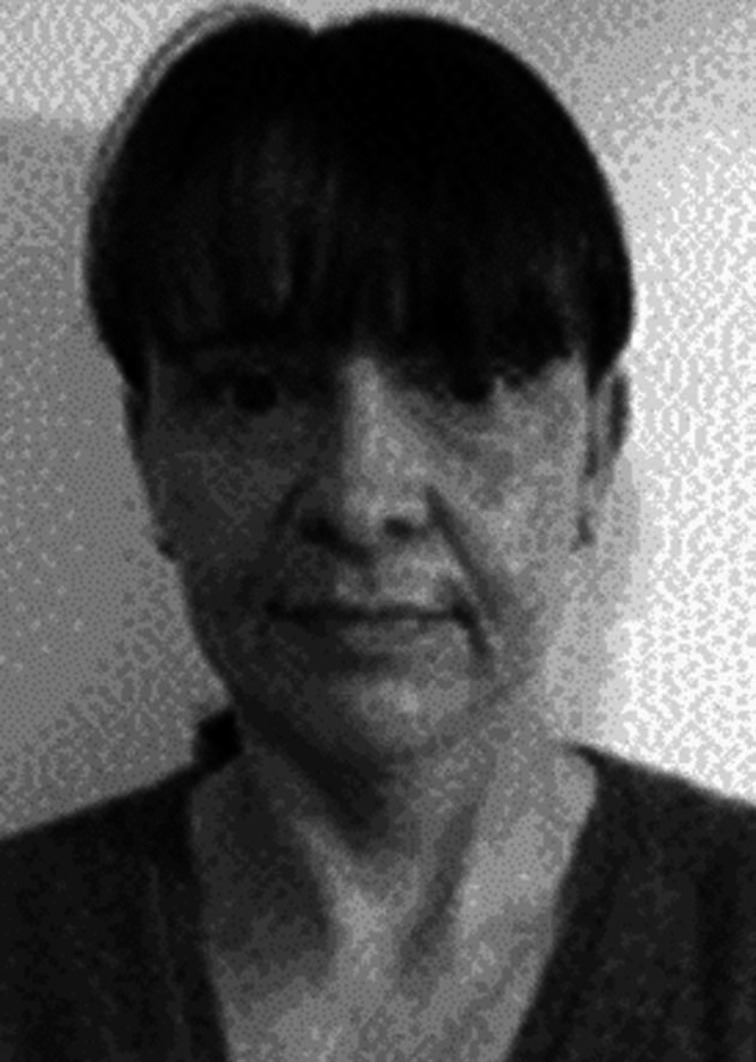# Synthetic Routes for Designing Furanic and Non Furanic Biobased Surfactants from 5‐Hydroxymethylfurfural

**DOI:** 10.1002/cssc.202200181

**Published:** 2022-04-22

**Authors:** Alexandra Velty, Sara Iborra, Avelino Corma

**Affiliations:** ^1^ Instituto de Tecnología Química Universitat Politècnica de València Consejo Superior de Investigaciones Científicas Avenida de los Naranjos s/n Valencia E-46022 Spain

**Keywords:** 3-hydroxycyclopentanol, 5-hydroxymethylfurfural, aromatics, levulinic acid, surfactants

## Abstract

5‐hydroxymethylfurfural (HMF) is one of the most valuable biomass platform molecules, enabling the construction of a plethora of high value‐added furanic compounds. In particular, in the last decade, HMF has been considered as a starting material for designing biobased surfactants, not only because of its renewability and carbon footprint, but also because of its enhanced biodegradability. This Review presents recent examples of the different approaches to link the hydrophilic and lipophilic moieties into the hydrophobic furan (and tetrahydrofuran) ring, giving a variety of biobased surfactants that have been classified here according to the charge of the head polar group. Moreover, strategies for the synthesis of different non‐furanic structures surfactant molecules (such as levulinic acid, cyclopentanols, and aromatics) derived from HMF are described. The new HMF‐based amphiphilic molecules presented here cover a wide range of hydrophilic‐lipophilic balance values and have suitable surfactant properties such as surface tension activity and critical micelle concentration, to be an important alternative for the replacement of non‐sustainable surfactants.

## Graphical abstract

**Amphiphiles**: In the last decade, 5‐hydroxymethylfurfural (HMF) has been considered as a starting material for designing biobased surfactants, not only because of its renewability and carbon footprint, but also because of its enhanced biodegradability. This Review summarizes recent examples of different approaches to provide new highly biodegradable biobased amphiphiles starting from HMF and its derivatives, which have been classified according to the charge of the head polar group.

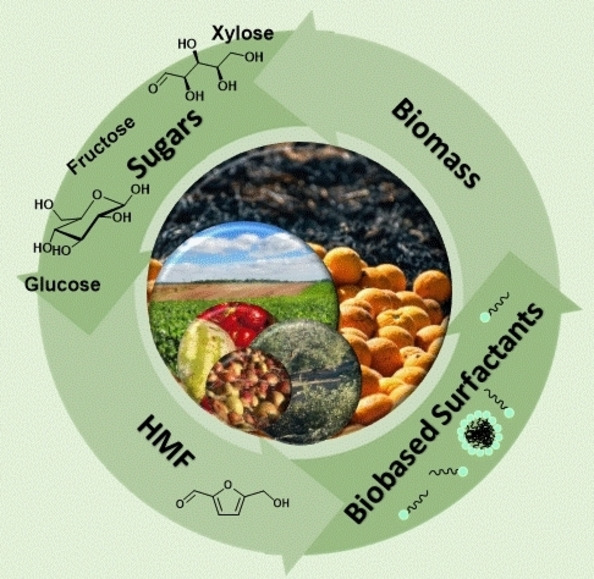

## 1 Introduction

The decision taken by our society to move towards decarbonization has made the chemical industry focus their interest on the use of biomass as a raw material and renewable feedstock as strong and sustainable alternative to products derived from fossil carbon.^[1]^ Carbohydrates represent about 75 % of the approximately 180 billion tons of biomass produced each year by nature. Cellulose can be readily depolymerized into hexoses through acid‐catalyzed hydrolysis and so, glucose can be considered an Earth‐abundant and cheap biomass raw material for chemical production. Thus, carbohydrates and platform molecules derived from it are intensively explored as feedstock for preparation of chemicals and fine chemicals.[^1^, ^2^, ^3^, ^4^, ^5^]

**Figure 1:**
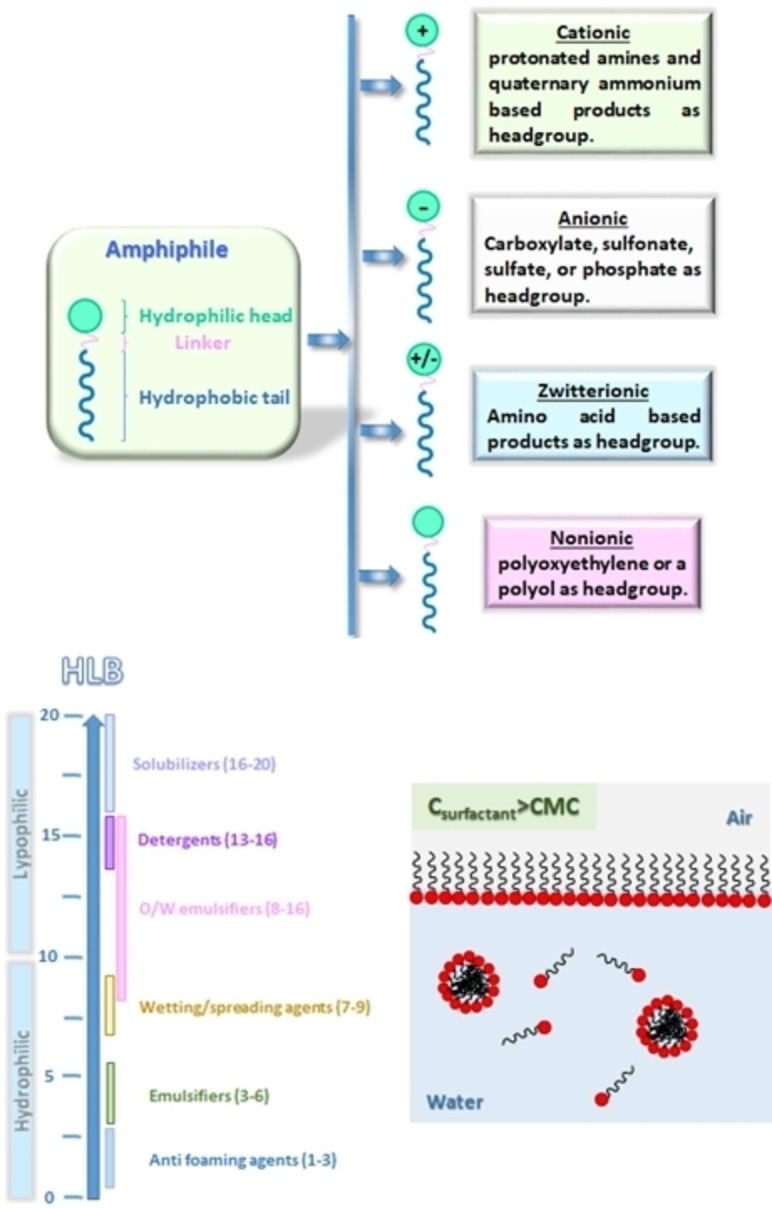
Different classes of surfactants. HLB scale, and representation of micelle formation at high concentration of surfactant in water.

Surfactants are amphiphilic molecules able to lower the surface tension between two liquids, and they find application in our daily life and in numerous sectors of the industry such as household and industrial detergents, cosmetics, paper, inks, coatings, food, and agrochemicals. Surfactants contain at least one hydrophilic ‘‘head’’ group and one lipophilic ‘‘tail” (Figure 1). The hydrophilic head group is commonly associated to carboxylic acid, sulfonic acid, sulfuric acid, amino group, and their salts, and also to nonionic polar groups such as hydroxy (sugar), amide, and ether (polyoxyethylene chain), while the lipophilic tail is conventionally a non‐polar alkyl chain (≥C_8_). A classification of surfactants is based on the charge of the head group when the amphiphile is dissolved in water, and they are cationic, anionic, amphoteric (or zwitterionic) compounds. Another classification is based on the ratio of the hydrophilic and hydrophobic portions of the molecule, which are determined by the hydrophilic‐lipophilic balance (HLB) value (Figure 1).[^6^, ^7^] The HLB value varies between 0 and 20, and it is a measure that indicates the type of emulsion that the surfactant forms (e. g., water‐in‐oil or oil‐in‐water), and can be used to predict the properties of a surfactant. The addition of a surfactant into a two‐phase system leads to surfactant adsorption at the liquid–liquid interface until saturation, and when concentration is increased, surfactant molecules can aggregate into large groups to form assemblies (micelles). The critical micelle concentration (CMC) is the minimum surfactant concentration necessary to initiate micelle formation (Figure 1). The CMC is usually used to measure the surfactant efficiency. For example, a good surfactant can lower the surface tension of water from 72 to 30 m Nm^−1^. Thus, more efficient surfactants have a lower CMC.^[8]^

**Scheme 1:**
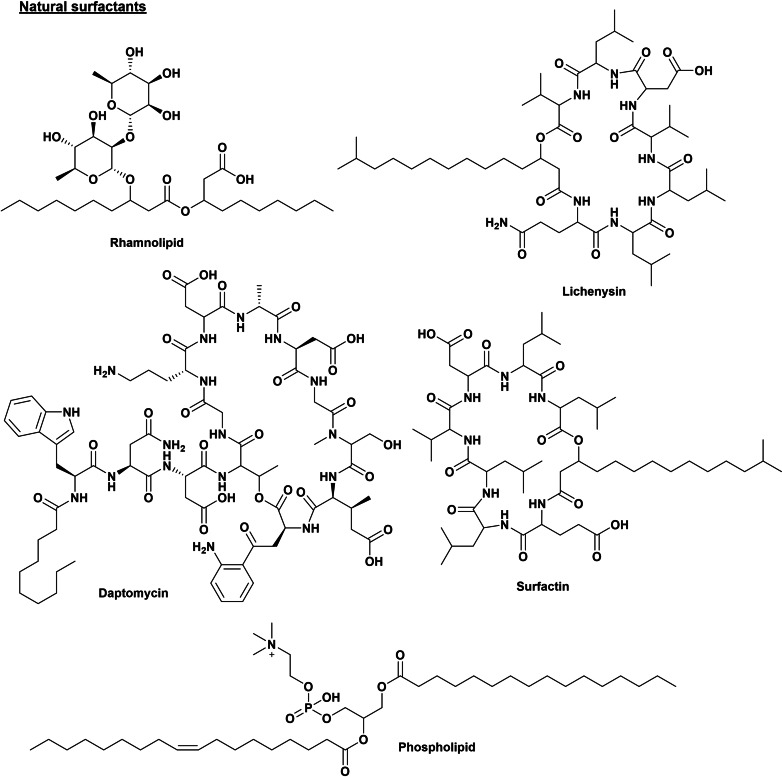
Representation of some biosurfactants.

**Scheme 2:**
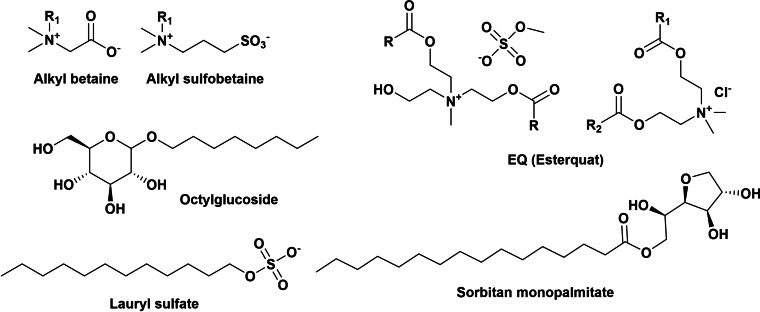
Representation of some commercial biobased surfactants.

The global surfactants market size was valued at $ 41.3 billion in 2019 and is expected to reach $ 58.5 billion by 2027.^[9]^ In the last decade, in view of the high demand for surfactants and the sustainable development goals, there has been a continuously growing interest in designing and synthesizing new renewable and degradable surfactants to limit their environmental impact. Renewable surfactants include surfactants produced by fermentation (microbes), also named biosurfactants (such as glycolipids, lipopolysaccharides, lipopeptides, phospholipids, and fatty acids; Scheme 1),^[10]^ and biobased surfactants where the head group or tail are based on renewables sources such as oleo‐chemicals, sugars (glucosides),[^11^, ^12^, ^13^, ^14^] or amino acids. In Scheme 2, some representative examples of biobased commercial surfactants are displayed.

**Scheme 3:**
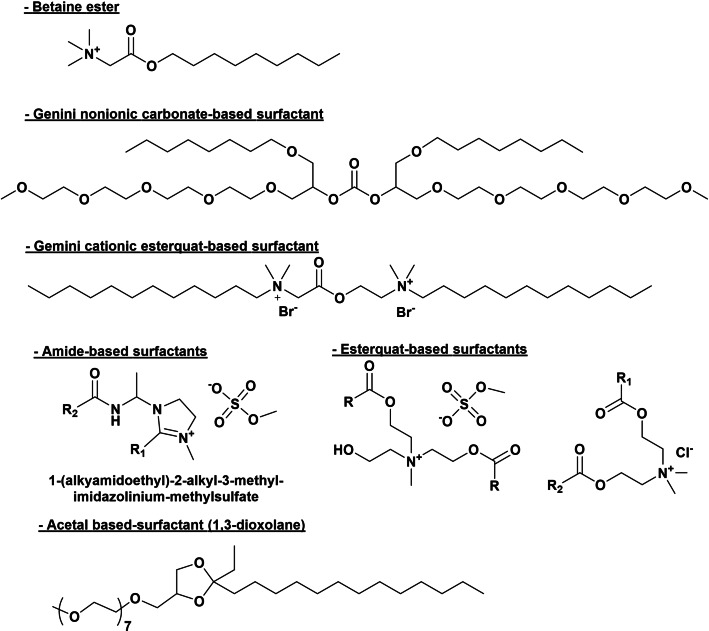
Examples of cleavable surfactants.

Due to the wide use of surfactants as industrial and domestic detergents, they are a main constituent of municipal and industrial wastewater,[^15^, ^16^, ^17^] sea and ocean (due to their use in oil spill remediation),[^18^, ^19^] and soil and plants.[^20^, ^21^] Surfactants are among the main environmental pollutants and, due to their interaction with cell membranes, they are biologically active and can alter essential biochemical processes and affect the well‐being of living organisms.[^22^, ^23^] The imperative control of surfactant concentration in the environment at minimum led to several regulations (in US and EU) stipulating, for example, that surfactants used in detergents must be fully biodegradable. Thus, to promote sustainability, manufacturers work hard to reduce the environmental impact of surfactants and focus on the use of biodegradable ingredients. A surfactant is considered to be biodegradable when it breaks down and decomposes into products found in Nature within a short time and therefore does not persist in the environment.^[24]^ A strategy to improve biodegradability (rate of biodegradation) of surfactants is to insert into their structure a labile bond that usually connects the polar head and the hydrophobic tail and that can be easily cleaved under chemical conditions (acid, alkali, UV light, heat, ozone) or by enzymes, implying the immediate destruction of the surface activity of the amphiphilic molecule (called cleavable, triggerable, or destructible surfactants).[^25^, ^26^, ^27^, ^28^, ^29^] In Scheme 3, some examples of cleavable surfactants are displayed.

Although the biodegradability of a material is independent of the origin of the starting raw materials used, the use of biodegradable natural raw materials (sugars, starch, oil, fat, glycerol) and derivatives for the synthesis of surfactants constitutes an alternative for obtaining potentially biodegradable surfactants.

Particularly, 5‐hydroxymethylfurfural (HMF), produced by dehydration of hexoses (mainly fructose), is one of the top biobased chemicals from carbohydrate biomass. HMF is a key platform molecule that enables the construction of a plethora of high value‐added furanic compounds for the production of bulk materials, fine chemicals, polymers, and also biofuels.[^30^, ^31^, ^32^, ^33^, ^34^, ^35^] In the last decade, HMF has also been considered as starting material for biobased surfactants not only due to its renewability and carbon footprint but also for their improved biodegradability. In the present report, we will focus on the different strategies used for the synthesis of new renewable and degradable biobased surfactants starting from HMF and its non‐furanic derivatives. Thus, the Review includes, in a first part, different processes to construct amphiphilic molecules bearing furan or tetrahydrofuran moieties, classified by the type of surfactant obtained (cationic, anionic, non‐ionic, and amphoteric). In the second section, strategies for the synthesis of different non‐furanic structure surfactant molecules derived from HMF will be described.

## 2 Biobased Oleofuran and Tetrahydrofuran Surfactants from HMF

### 2.1 Anionic surfactants

**Scheme 4:**
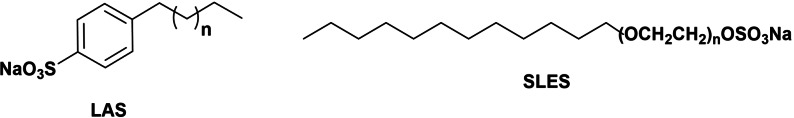
Examples of commercial sulfonates and sulfates anionic surfactants.

Anionic surfactants are widely used in the manufacture of industrial and household cleaning products due to their foaming capacity, ability to stabilize water‐in‐oil emulsions, and their ability to bind and remove positively charged particles, such as clay, dirt, and stains, by ionization. Linear alkylbenzene sulfonates (LAS) (Scheme 4) are the oldest anionic surfactants and most widely used as main components in the detergent composition, personal, and household care products. LAS toxicity depends on the length of the alkyl chain and the substitution position; the longer the alkyl chain the greater the toxicity.[^36^, ^37^] Alkyl ether sulfates (AES) are another group of surfactants largely used in laundry and personal care products. Among them, sodium lauryl ether sulfate (SLES) (*n*‐C_12_H_25_(OCH_2_CH_2_)_*n*_OSO_3_Na) is the most important AES, and also the main component of soil conditioning products used in the excavation industry and fracking.

**Scheme 5:**
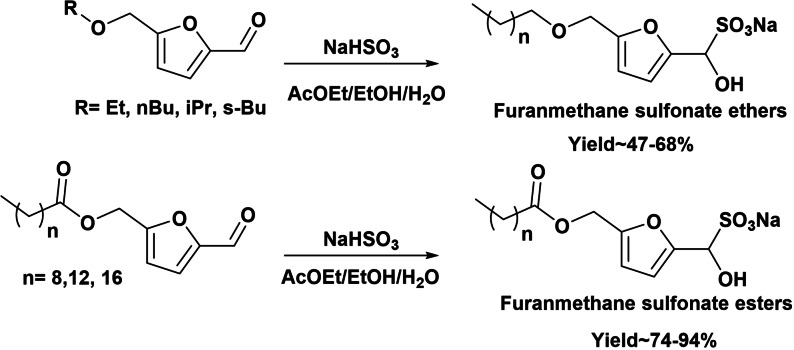
Preparation of furanmethane sulfonate esters and ethers.

In the last years, the need to prepare chemicals from renewable materials to face environmental concerns over fossil fuels consumption has motived the preparation of new oleofuran sulfonates from renewables with strong surfactant performance in minimal concentrations.[^38^, ^39^] Thus, due to structural similarities with linear alkylbenzene sulfonates, furanmethane sulfonate esters and ethers can be an interesting alternative to the fossil‐sourced LAS. Kraus and Lee^[40]^ selectively prepared furanmethane sulfonate esters and ethers by reacting suitable aldehydes with an ether or ester alkyl tail, with a mild sulfonating agent such as sodium bisulfite, preserving the ester/ether moiety and yielding 47–68 % ethers and 74–94 % esters (Scheme 5).

**Scheme 6:**
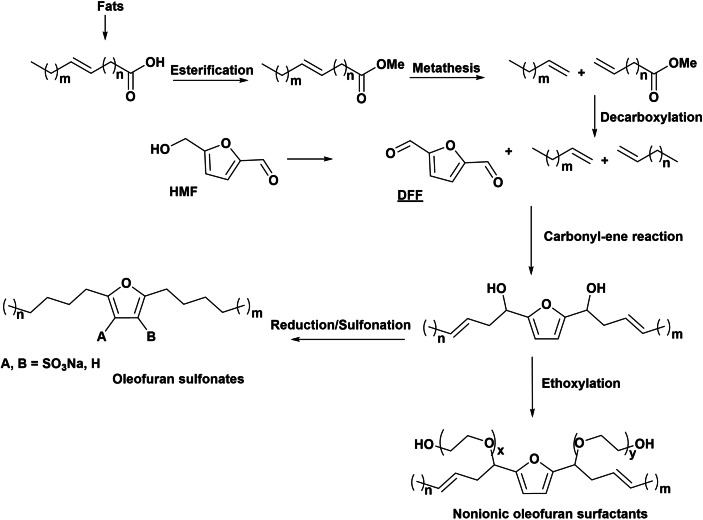
Preparation of oleofuran sulfonates as new anionic surfactants.

A very different route to design renewable oleofuran sulfonate molecules has been recently patented.^[41]^ The preparation of these new amphiphiles is based on carbonyl‐ene reaction between 2,5‐diformylfuran (DFF) and olefins issued from metathesis of renewable fatty acids and further sulfonation of the furan ring (Scheme 6). Olefins could be produced by initial esterification of fatty acid, followed by olefin metathesis in the presence of transition metal alkylidine complexes and zeolite‐supported transition metal oxides, leading to two terminal alkenes. After decarboxylation, in the presence of metal coordination complexes such as Ru_3_(CO)_12_, supported metal catalysts (Ni, Co, Cu, Pd, and Pt) on carbon, metal oxides, and mixed metal oxides, the olefins undergo carbonyl‐ene reaction with DFF to produce dialkyl furan compounds with two alcohol groups in α‐position of the furan ring. Therefore, one or more hydrophilic functional groups can be added by sulfonation to provide new amphiphilic molecules with new potential activity as anionic oleofuran surfactants (Scheme 6). Additionally, instead of sulfonation, the molecule can be ethoxylated through the alcohol groups, leading to a new non‐ionic surfactant.

**Scheme 7:**
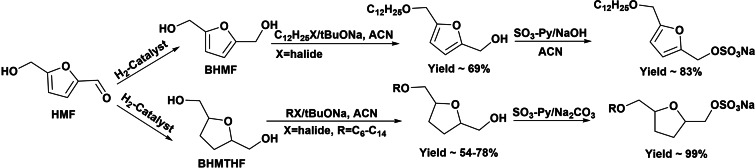
Preparation of new biobased sulfate surfactants from BHMF and BHMTHF.

The preparation of new biobased sulfate surfactants starting from renewable 2,5‐bis(hydroxymethyl)furan (BHMF) and bis(hydroxymethyl)‐tetrahydrofuran (BHMTHF), produced by HMF hydrogenation, was claimed as possible replacements of LAS and AES.[^42^, ^43^] Then, starting from BHMF and BHMTHF, long‐chain alkyl ether derivatives were synthesized by Williamson reaction that were subsequently sulfated with SO_3_‐Py complexes giving yields up to 97 and 80 % of the sulfate salts, respectively (Scheme 7).^[42]^ However, with chlorosulfonic acid as sulfating agent considerably lower yields were obtained (43 and 24 %) for BHMF and BHMTHF sulfate derivatives, respectively.^[43]^

The evaluation of the surfactant properties of these compounds with different chain lengths revealed that longer hydrophobic chains (C_12_–C_14_) allowed lowering CMC (0.02–0.05 g L^−1^) and interfacial tension (2.3–4 m Nm^−1^) towards isopropyl myristate, similarly to LAS, with CMC around 0.08 g L^−1^ and surface tension around 0.9 m Nm^−1^, and AES, with CMC around 0.05 g L^−1^ and surface tension around 3.9 m Nm^−1^, showing high potential for use in detergents, dispersants, and plasticizers.

**Scheme 8:**
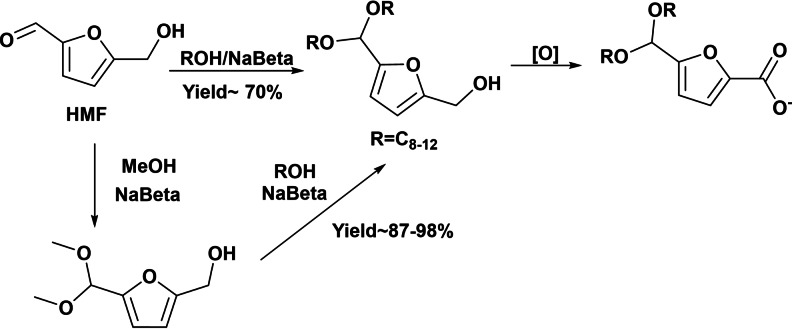
Preparation of anionic surfactant by successive acetalization‐transacetalization with fatty alcohol and oxidation reaction.

In addition to oleofuran sulfonates and sulfates, the synthesis of anionic surfactants based on oleofuran carboxylates has been recently reported. One of the first examples was the preparation of anionic biodegradable surfactants by coupling HMF and fatty alcohols using heterogeneous acid catalysts. The introduction of the hydrophobic tail in the HMF molecule was firstly achieved through the acetalization of the formyl group of HMF with fatty alcohols to obtain a cleavable anionic surfactant precursor molecule in the presence of a suitably designed Beta zeolite.^[44]^ Since Beta zeolite is able to competitively catalyze the acetalization of aldehyde group and etherification of the hydroxymethyl group of HMF, the control of Beta zeolite acidity by partial Na^+^ exchange was required to selectively perform the acetalization of HMF with *n*‐octanol (Scheme 8). The study of direct acetalization between HMF and *n*‐octanol revealed that despite the use of high ratio *n*‐octanol/HMF and the optimization of acidity and polarity of Beta zeolite, 70 % maximum yield of dioctyl acetal of HMF was obtained. To improve the yield, a process consisting of a one‐pot two‐step acetalization‐transacetalization reaction was designed. For that, dimethylacetal of HMF was prepared in the first step in the presence of the zeolite catalyst, and then the dimethyl acetal was transacetalized with *n*‐octanol (Scheme 8). The catalytic study showed that a partial Na‐exchanged Beta (NaBeta) zeolite was an efficient catalyst to perform both acetalization and transacetalization in a one‐pot process. Using this strategy, 95 % yield of dioctyl acetal of HMF was obtained. The one‐pot process was successfully extended to other alcohols with C_6–12_ chains yielding the corresponding dialkyl acetals with high selectivity (>96 %; see Table 1), and the NaBeta catalyst was recycled without loss of activity or selectivity.

**Table 1:** Results of the synthesis of alkyl acetals from HMF by one‐pot process using NaBeta zeolite as catalyst.^[a]^

Entry	Alcohol	*t* [h]	Conv. [%]	Yield of dialkyl acetal [%]	Sel. to dialkyl acetal [%]
1	hexanol	0.5	100	98	99
2	octanol	0.5	100	95	98
3	decanol	2	93	90	97
4	dodecanol	2	90	87	96

[a] Reaction conditions: step 1: HMF (1 mmol), MeOH (123 mmol, 5 ml), NaBeta (26 mg), at 65 °C, 2.5 h; step 2: alcohol (3.7 mmol) at 65 °C.

To introduce the hydrophilic moiety of the surfactant, a second step involving the oxidation of the hydroxymethyl group into carboxylic acid and neutralization can be performed to obtain 5‐(dialkoxymethyl)‐2‐furoate surfactants. The high performances of the process using stable and reusable solid acid catalyst, along with the fact that solvents or additives are not required, represent a promising sustainable route to produce these cleavable surfactants that should then show good biodegradability.

**Scheme 9:**
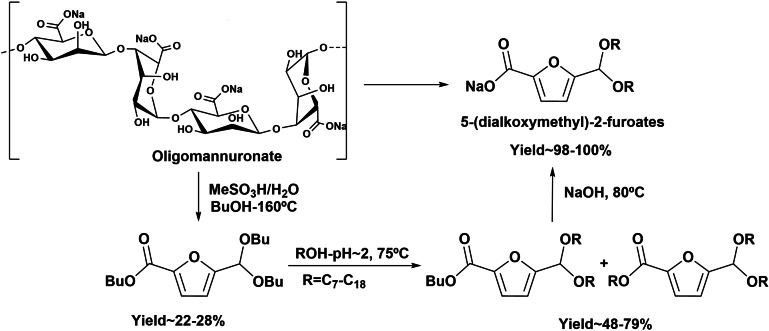
One‐pot synthesis of 5‐(dialkoxymethyl)‐2‐furoates from oligomannuronates.

Recently, an alternative route towards the anionic sodium 5‐(dialkoxymethyl)‐2‐furoates starting directly from alginate or oligo‐alginates (Scheme 9) through a one‐pot process has been reported.^[45]^ In this process, butyl 5‐(dibutoxymethyl)‐2‐furoate is formed *in situ* by cyclodehydration of *n*‐butyl glycoside uronate intermediates, under aqueous acid media in presence of butanol. The butyl 5‐(dibutoxymethyl)‐2‐furoate intermediate is transacetalized with a fatty alcohol and subsequently saponified, yielding the target product. This one‐pot cascade procedure yielded acetal‐based furanic carboxylate surfactants with 15–20 % molar yield with respect to oligomannuronates. The evaluation of surfactant properties of sodium 5‐dioctylacetal‐2‐furoates such as CMC, surface tension activity towards water, and foaming capacity, revealed that this compound presents a CMC and surface tension of 0.31 g L^−1^ and 31.7 m Nm^−1^, respectively, exhibiting promising properties as anionic surfactants able to replace the conventional fossil‐based SLES with CMC and surface tension around 0.33 g L^−1^ and 31.8 m Nm^−1^, respectively. Moreover, a study of biodegradability showed that sodium 5‐(dioctyloxymethyl)‐2‐furoate exhibited ready biodegradability and absence of aquatic ecotoxicity.

**Scheme 10:**
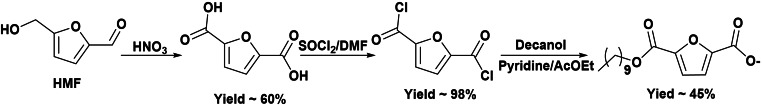
Preparation of decyl FDCA monoester as biodegradable surfactant.

The preparation of decyl furan dicarboxylic acid monoester (decyl‐FDCA ester) bearing a hydrophilic carboxylate head group and fatty ester as lipophilic tail as biodegradable surfactant was recently described starting from HMF.^[47]^ First, the oxidation of HMF with HNO_3_ into 2,5‐furandicarboyxlic acid (FDCA) was carried out, which was converted into 2,5‐furandicarbonyl dichloride by reaction with SOCl_2_. Selective mono‐esterification of the dichloride derivative with *n*‐decanol gives the decyl‐FDA monoester (Scheme 10). The biodegradability of the decyl ester was explored according to the OCDE 301F standard, based on the biological oxygen consumption and the calculation of the theoretical oxygen consumption. The decyl‐FDCA monosodium salt showed a 68 % final level of biodegradability and was considered to be biodegradable. However, from environmental and industrial standpoints, a greener process for their production should be explored.

**Scheme 11:**
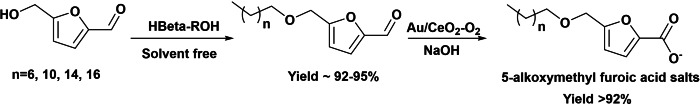
Sustainable and green preparation of 5‐alkoxymethyl furoic acid salts as renewable and biodegradable surfactants.

Another interesting type of surfactants based on furoic acid salts was produced by introducing an ether moiety (instead of an ester) as lipophilic part. The process involves the selective etherification of the hydroxymethyl group of HMF with fatty alcohols in the presence of solid acid catalyst, followed by the oxidation of the formyl group into carboxylate sodium salt (Scheme 11).^[48]^ The etherification step was explored in the presence of protonic zeolites with different topologies and variable adsorption/desorption properties. The catalytic study revealed the suitable catalytic properties of hydrophobic‐defect free H‐Beta zeolites with Si/Al ratios higher than 25, which exhibited excellent activity to carry out the selective etherification of HMF with fatty alcohols, minimizing the competitive self‐etherification of HMF (<3 %). Excellent yield of the fatty ethers regardless of the chain length (C_8_–C_18_) were obtained at 100 °C in a few hours. Then, the 5‐alkoxymethylfurfural compounds were selectively oxidized into the corresponding sodium furoic salts with excellent yield over Au/CeO_2_ using air as oxidant, in a basic aqueous solution at 65 °C. It was shown that both catalysts (H‐Beta zeolite and Au/CeO_2_ samples) were stable and could be reused several times. The overall yield and selectivity of the two combined steps for the production of biodegradable and renewable anionic surfactants with the different fatty alcohols (C_8_–C_18_) were very high (Table 2), which demonstrated the high efficiency and sustainability of the developed catalytic process. Measurements of the surface tension in water solutions of these surfactants showed that 5‐(octadecyloxymethyl)‐2‐furoic acid sodium salt (SODF) had a similar surface tension (35 m Nm^−1^) as the commercial dodecylbenzene sulfonic acid sodium salt (SDBS) (33 m Nm^−1^) for 1 wt% concentration in water, although the CMC for SODF was 0.9 g g^−1^ while reported CMC^[49]^ for SDBS was 0.413 g L^−1^.^[48]^

**Table 2:** Results of the conversion of HMF into of 5‐alcoxymethylfurfural derivatives through the etherification‐oxidation steps.

Entry	Alcohol	Conv. [%]	Yield of 5‐alcoxy‐methyl furoic acid [%]	Sel. [%]
1	octanol	100	92	92
2	dodecanol	100	94	9
3	hexadecanol	100	95	95
4	octadecanol	100	95	95

Reaction conditions: step 1: HMF (1 mmol), *n*‐alcohol (1 mmol), 100 °C, HBeta (Si/Al=12.5) catalyst (40 wt% with respect to HMF); step 2: H_2_O (14 mL) with NaOH (molar ratio NaOH/HMF=1) Au/CeO_2_ (molar ratio HMF/Au=78), air (10 bar), 65 °C.

### 2.2 Cationic surfactants

**Scheme 12:**
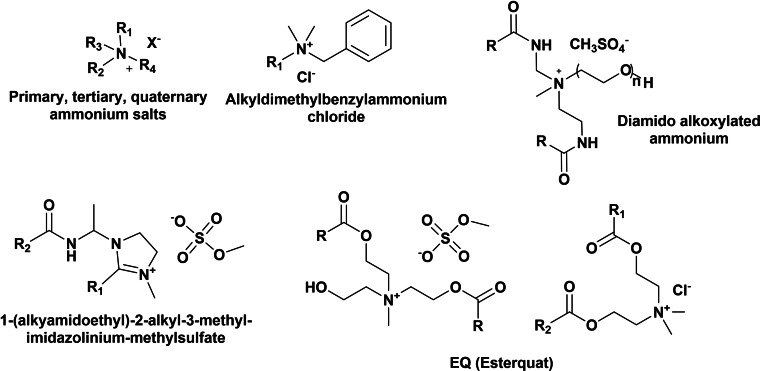
Ammonium‐based amphoteric and cationic surfactants as detergents components.

Cationic surfactants are usually quaternary ammonium salts bearing long‐chain alkyl groups (C_16–22_), sometimes branched, usually used as ingredients in softeners, haircare products, anti‐corrosion products and as dispersants and emulsifiers. If anionic surfactants are the most important ingredients in detergents due to their cleaning efficiency, cationic surfactant are the main components in softeners being distearyldimethylammonium chloride (C_18_ chain), the first worldwide cationic softener produced.^[50]^ The main chemical classes of cationic surfactants are primary, tertiary, and quaternary ammonium salts, and among them benzyl quaternary ammonium salts, imidazolinium compounds, diamido alkoxylated ammonium, and esterquats derived from ethanolamine are the most important classes of cationic surfactants (Scheme 12). Accordingly, to their chemical structure and nature, they have a pH‐dependent positive charge.

**Scheme 13:**
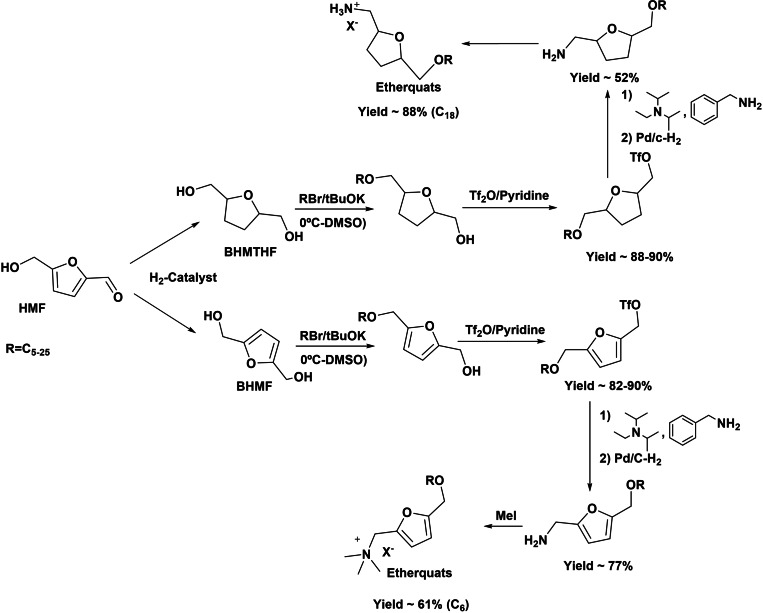
Preparation of new cationic biobased surfactants from BHMF and BHMTHF.

Furan‐containing quaternary ammonium salts have been recently reported in alternative production of renewable new ion pairs with application as ionic liquid solvents, surfactants, or biocides in analogy with benzyl quaternary ammonium salts.^[51]^ A recent patent^[43]^ describes the preparation of new cationic biobased surfactants from BHMF and BHMTHF. The synthetic process involves first the direct preparation of linear monoalkyl ethers from the BHMF and/or BHMTHF through the Williamson reaction. The monoalkyl ethers are subsequently converted into triflate derivatives by reaction with triflic anhydride, which after substitution with an amine is converted into a furfuryl (or tetrahydrofurfuryl) amine derivative and subsequently quaternized into an ammonium salt (Scheme 13). The synthesized compounds form a new class of renewable etherquats that can be used as surfactants, dispersants, and plasticizers.^[43]^

**Scheme 14:**
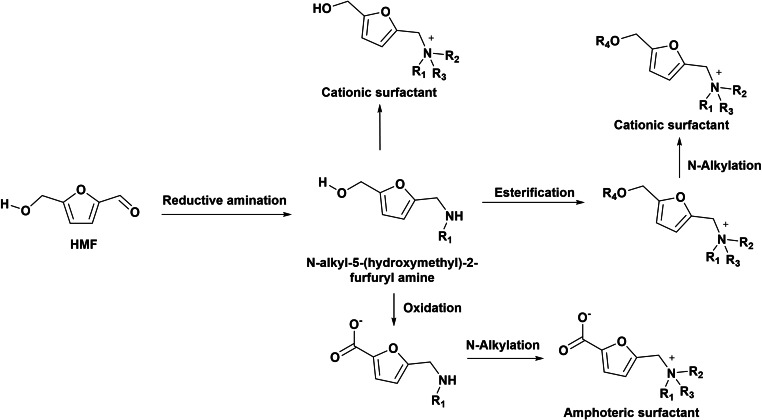
*N*‐substituted 5‐(hydroxymethyl)‐2‐furfuryl amines as precursors of biobased amphoteric or cationic surfactants.

As showed above, the synthesis of quaternary ammonium salts starting from BHMF and BHMTHF requires the formation of a triflate derivative precursor, in which the triflate group is subsequently displaced by an amine giving the furfurylamine derivative. Although this route gives acceptable yields of the target compound, it suffers of low atom economy. An alternative method for the formation of furfurylamine derivatives (precursors of the quaternary ammonium salts) can be directly achieved by reductive amination of the formyl group of HMF (or derivatives) with ammonia or primary amines (Scheme 14). The reaction involves as first step the formation of an imine intermediate that is subsequently hydrogenated into amine using reducing agents such as NaBH_4_ or hydrogen.

**Scheme 15:**
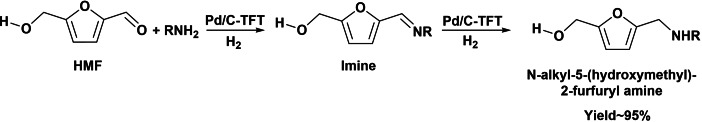
Reductive amination of HMF for *N*‐substituted 5‐(hydroxymethyl)‐2‐furfuryl amines synthesis over Pd/C catalysts.

A recent Review compiled the latest advances for the reductive amination of HMF focused on the use of H_2_ in the presence of homogeneous and heterogeneous metal‐based catalysts or in the presence of sodium borohydride as reductant.^[52]^ Accordingly, Ru‐based complex catalysts were used under H_2_ pressure, in ethanol, by direct reductive amination of HMF with primary and secondary amines to produce *N*‐substituted aminomethyl‐hydroxymethylfurans with good yields up to 95 %.^[53]^ However, considering the current trend and the need to develop sustainable and environmentally friendly processes, the use of efficient, robust, and recyclable heterogeneous catalysts is highly desired and of great interest. Thus, *N*‐alkyl‐5‐(hydroxymethyl)‐2‐furfuryl amine surfactant precursors were obtained in excellent yields by reductive amination of HMF with fatty amines in a one‐pot two‐step process using hydrogen and Pd supported catalyst.^[54]^ Interestingly, it was shown how the nature of the support influences the distribution of metal active sites that in turn has an impact on the furfurylamine selectivity. Thus, the Pd nanoparticles supported on activated carbon (1 % Pd/C) gave total selectivity to the corresponding amine while avoiding the further hydrogenation of the furan ring. This behavior was attributed to the deposition of carbonaceous species on the Pd terraces (where furan ring hydrogenation mainly occurs) increasing the proportion of unsaturated Pd sites, which are active for selective hydrogenation of the C=N group (Scheme 15).^[54]^ Moreover, a study of catalyst stability and recyclability revealed that the Pd/C sample maintained high activity over three successive runs. This strategy represents an efficient, simple, and sustainable atom‐economical route for producing *N*‐substituted furfurylamine precursors of surfactants based on furan‐quaternary ammonium salts.

### 2.3 Nonionic surfactants

Nonionic surfactants are the second class of surfactants more used for industrial and household cleaning products, cosmetics, and disinfectants. They contain as hydrophilic moiety non‐water ionizable groups such as polyoxyethylene, ether, polyhydroxyl, amide, and so on, giving neutral dissolutions. They exhibit higher emulsifying capacity for oils than anionic surfactants for removal of organic soils/grease, and thus combinations of nonionic and anionic surfactants are frequent. The water solubility of the head group is due to hydrogen bonding, which decreases with increasing temperature, and consequently the water solubility of nonionic surfactants decreases with increasing temperature. The decrease in solubility leads to the formation of a cloudy emulsion called the cloud point. This property is unique to nonionic surfactants and is determining for their optimum use in formulations at high temperature. The cloud point depends on the ratio of hydrophilic/hydrophobic moieties and varies from room temperature to very high temperature; for instance, in the case of a very high hydrophilic/hydrophobic ratio, there is no cloud point.

**Scheme 16:**
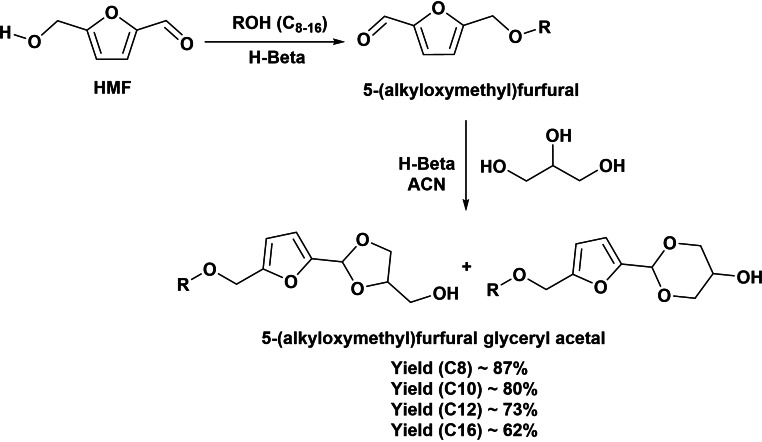
Preparation of new nonionic biobased surfactants with HLB 4.9–6.6.

Recently, we have reported the preparation of new nonionic biobased amphiphilic molecules starting from three renewable chemicals (HMF, glycerol, and fatty alcohols) trough a one‐pot cascade process, leading to a family of interesting cleavable surfactants with high sensitivity to acidic conditions (Scheme 16).^[55]^ The process involves as first step the etherification of HMF with fatty alcohols to introduce the hydrophobic moiety, giving the 5‐alkoxymethyl furfural intermediate that is subsequently acetalized with glycerol into cyclic acetal isomers (1,3‐dioxolanes and 1,3‐dioxanes) (hydrophilic moiety). Both steps are performed in the same reaction vessel using an optimized Beta zeolite of high Si/Al ratio free of connectivity defects (Scheme 16). Following this strategy, a variety of new cleavable surfactant molecules with HLB values in the range 4.9 to 6.6 were obtained with excellent yields. The HLB values indicate that these new surfactants can be used for water‐in‐oil emulsions, similarly to sorbitan monoesters such as sorbitan stearate and sorbitan monopalmitate (SPAN 60, SPAN 40).

**Scheme 17:**
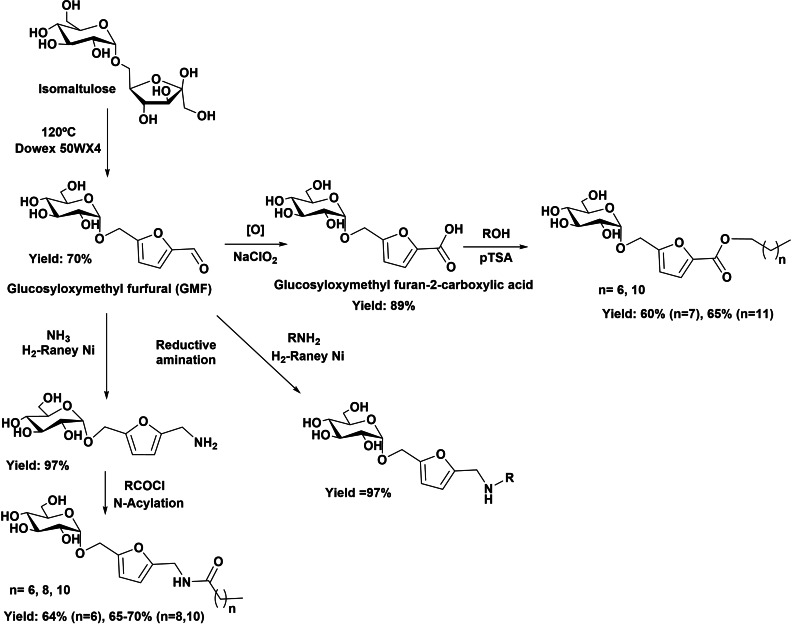
Preparation of new nonionic oleofuran surfactants from isomaltulose.

Cleavable acetal linkages have been also used in the preparation of glucosilated HMF derivatives (Scheme 17).^[56]^ Dehydration of isomaltulose leads to glucosyloxymethylfurfural intermediates (GMF); this polar moiety is linked to a hydrophobic tail through the formyl group following different strategies: (i) oxidation of the formyl group of GMF giving glucosyloxymethylfuran‐2‐carboxylic acid, which is esterified with fatty alcohols to provide GMF‐esters; (ii) reductive amination of GMF with ammonia that provides a primary amine, which is subsequently converted into amide by reaction with a fatty acid chloride; and (iii) reductive amination with fatty amines. The evaluation of the physicochemical properties of these compounds revealed that GMF‐amides compounds had CMC close to 1.5 mmol L^−1^ while in the GMF‐esters compounds the CMC was in the range 0.06–0.08 mol L^−1^, supporting their potential as new nonionic surfactants.

**Scheme 18:**
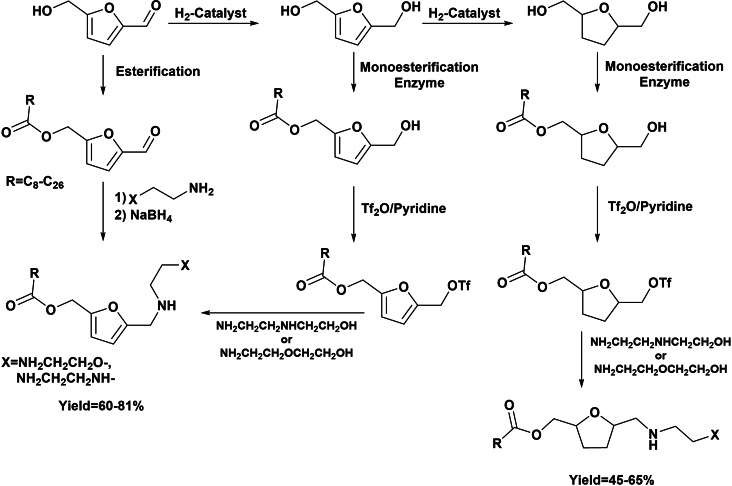
Preparation of non‐ionic green surfactants.

A recent patent describes the tedious preparation of nonionic surfactants with polar head group based on furan‐substituted diaminodiethylamine or aminoethylethanolamine and fatty monoester as hydrophobic tail, starting from HMF, BHMF, and BHMTHF.^[57]^ The renewable precursors was first monoesterified in the presence of a lipase (Candida Antartica B, Lipozyme 435) with a fatty acid (C_8_–C_26_). The further reductive amination of the HMF monoester with suitable amine using NaBH_4_ allowed obtaining new amphiphilic molecules bearing a functional group having sufficient hydrogen bonding capacity to provide hydrophilic properties to the furan moiety (Scheme 18). Starting from BHMF and BHMTHF, the sulfonation with anhydride triflate of the intermediate monoesters was required, and further addition of suitable amine allowed obtaining new amphiphilic molecules (Scheme 18). Besides the monoesterification in the presence of enzyme, a more environmentally friendly synthetic approach should be explored.

**Scheme 19:**
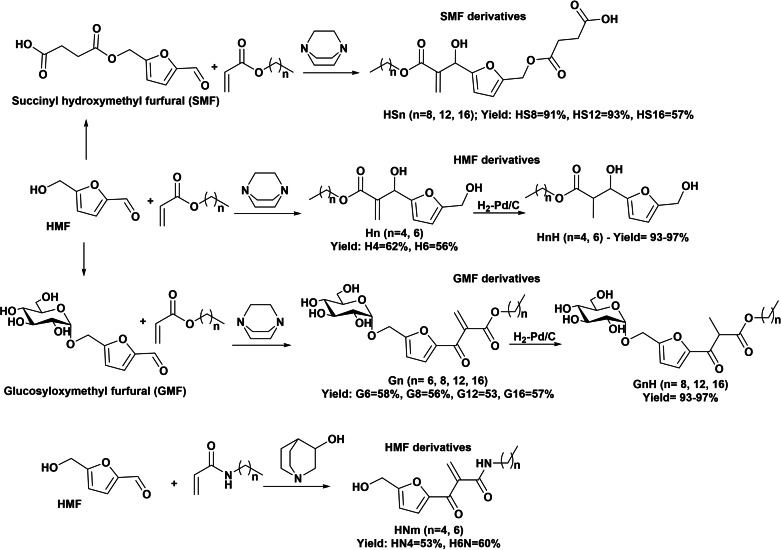
Preparation of new nonionic oleofuran surfactants by Morita–Baylis–Hillman reaction.

Very recently, the preparation of a series of new anionic surfactants by green Morita–Baylis–Hillman reaction via addition of hydrophobic α,β‐unsaturated carbonyl compounds (acrylates or acrylamides) to furanic aldehydes catalyzed by a tertiary amine was reported (Scheme 19).[^58^, ^59^, ^60^, ^61^] Glucosyloxymethyl furfural (GMF), succinyl hydroxymethyl furfural (SMF), and HMF constituted the polar head group and conferred different surfactant abilities to the amphiphilic molecules. The determination of the physicochemical properties revealed that these amphiphiles had in general low solubility in water. The α‐methylene‐β‐hydroxycarbonyl compound derivatives obtained directly from HMF (Hn compounds in Scheme 19) exhibited low solubility in water, which was improved after hydrogenation of double bond (HnH compounds). The presence of SMF moiety did not improve water solubility, while the glucose moiety in GMF derivatives provided enhanced water solubility. A study of the effect of hydrogenation of the double bond showed that the CMC was slightly higher for the saturated compounds (Gn) than for unsaturated ones (GnH) (1.3–1.8 times). This effect was attributed to the improved solvation of the double bond, unfavorable to micelle formation. No Krafft point was found, and the synthesized amphiphiles behave as nonionic surfactants. The evaluation of HLB by the PIT/slope method allowed comparing the HLB of the furanic surfactants with that of other nonionic surfactants. Thus, for instance, G12H exhibited HLB value close to dodecyl‐β‐d‐glucopyranoside and G8 to dodecyl‐β‐d‐glucopyranoside. Preliminary tests for cosmetic oil emulsification indicated that most GMF derivatives were able to stabilize oil‐in‐water (O/W) emulsions while SMF derivatives were able to stabilize water‐in‐oil (W/O) emulsions (H16S). This new series of biobased surfactants constitutes alternatives to the use of conventional polyethoxylated surfactants for applications in detergents and cosmetics.

### 2.4 Amphoteric and amphoteric gemini surfactants

**Scheme 20:**
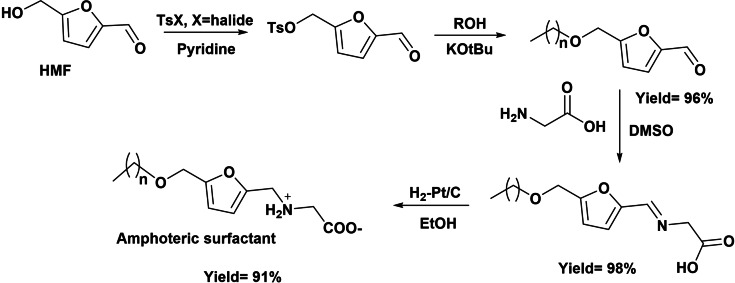
Preparation of new amphoteric biobased oleofuran surfactants.

Amphoteric surfactants have both positive and negative charged centers that maintain overall neutrality. However, their properties vary accordingly with the pH value and behave as cationic surfactant below the isoelectric point, as amphoteric surfactant close to the isoelectric point, and as anionic surfactant at higher pH. Due to their specific structure, amphoteric surfactants exhibit unusual properties such as high water solubility, high surface activity, low CMC, and high foaming capacity. Therefore, in the last decades, many investigations focus on their synthesis and uses in cosmetics, polymers, pharmaceuticals, detergents, and cleaning agents.[^29^, ^62^] Amphoteric surfactants have been prepared from HMF, which after derivatization into HMF tosylate can undergo etherification with fatty alcohols in the presence of potassium *t*‐butoxide to give an ether. The reductive amination of the 5‐alkoxymethylfurfural obtained with glycine leads to the target amino‐acid‐based amphiphile (Scheme 20).^[63]^

**Figure 2:**
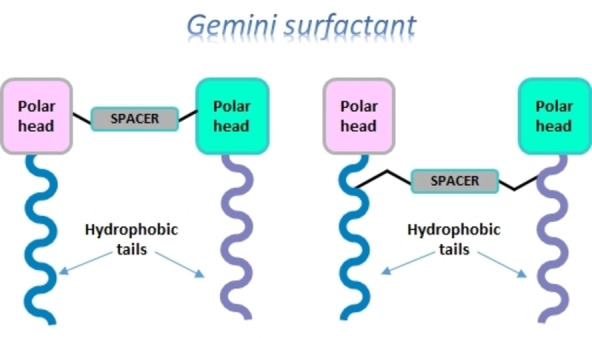
Representation of a gemini surfactant.

In the last two decades, the scientific community has focused on the unusual physicochemical properties of gemini surfactants, such as low CMC, water solubility, unique micelle structures (spherical, ellipsoidal, helical, tubular, rod shape, vesicles, and helical),^[64]^ aggregation behavior, high surface tension activity, and also rheological properties.[^64^, ^65^, ^66^, ^67^, ^68^, ^69^] Then, gemini surfactants were reported to be three orders of magnitude more effective in reducing water surface tension compared to traditional surfactants. Structurally, all gemini surfactants possess at least two hydrophobic chains and two polar groups that are connected by a flexible or rigid spacer. The nature of the head can be cationic, non‐ionic, anionic, or zwitterionic, and the tail is usually a short or long hydrocarbon chain while the nature of the spacer varies and can be polar or non‐polar (Figure 2). Owing to their extraordinary physicochemical and biological properties, gemini surfactants find a wide range of advanced applications as emulsifiers, dispersants, foaming, corrosion inhibitors, biocides, in solubilization, and in drug and gene delivery.

Therefore, a large variety of gemini surfactants can be prepared by changing the ionic and chemical structure of the head group, the nature of the spacer, the hydrophobic chain length, or the symmetry of the molecule in order to achieve superior surface adsorption, aggregation, phase behavior, antimicrobial activity, and biodegradation performance.

**Scheme 21:**
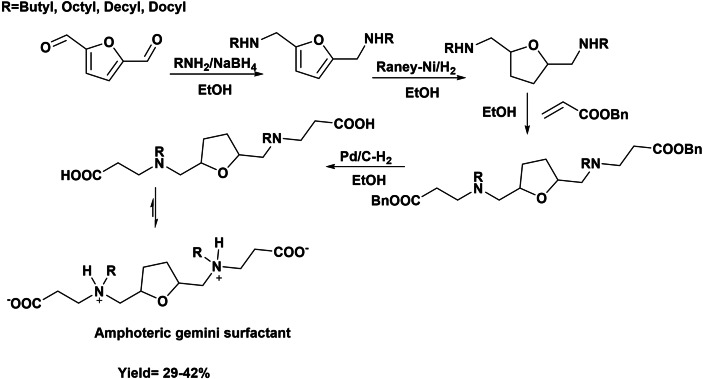
Preparation of amphoteric gemini surfactants from DFF.

Recently, a new family of amphoteric gemini surfactants has been synthesized starting from DFF. Structurally, they are formed by a tetrahydrofuran moiety as spacer, two β‐aminoacids as amphoteric heads (betaine moieties), and two alkyl chains as hydrophobic tails (Scheme 21).^[70]^ The synthesis involved first the preparation of a *N*‐substituted furfuryldiamine derivative by reductive amination of DFF with different fatty primary amines using NaBH_4_ as reductant. Then, the reduction of the furan ring with Raney‐Ni is performed to yield tetrahydrofuran intermediates. These compounds were further condensed with benzylacrylate to introduce the anionic group, and finally the protecting group of carboxylic acid was removed by hydrogenolysis over Pd/C catalyst generating betaine moieties (Scheme 23). The total yield of three‐step surfactants synthesis with C_4–12_ alkyl chain was between 29–42 %.

**Scheme 22:**
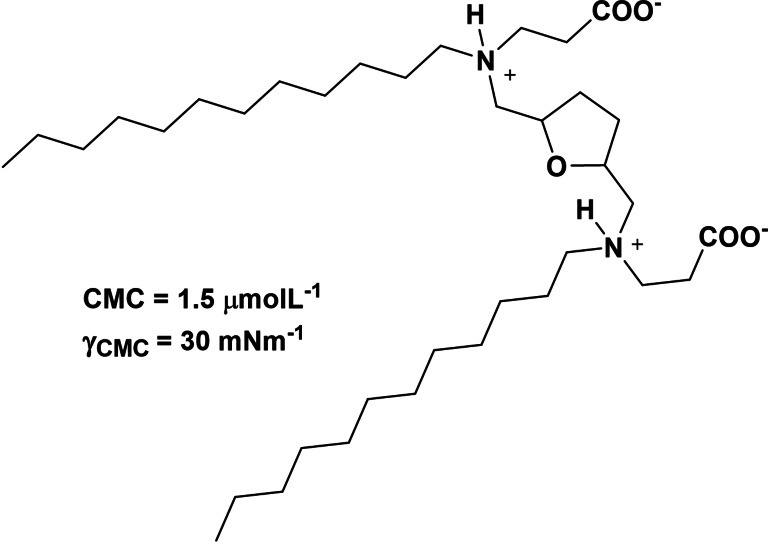
Synthesis of biobased gemini surfactant with low CMC and efficient surface tension activity.

The evaluation of surfactant properties such as the CMC and surface tension reduction of aqueous solution (pH=4–9) (Scheme 22) revealed very low CMC close to around 1.5 μmol L^−1^, which was considerably lower than that of gemini imidazoline amphoteric surfactants (2.25 mmol L^−1^), while the decrease of the surface tension (up to 30 m Nm^−1^) was similar to that gemini imidazoline amphoteric surfactant (30.42 m Nm^−1^)^[71]^ and alkylbetaine zwitterionic gemini surfactants aqueous solution.^[72]^ Moreover, a study of antifungal activity against Fusarium graminearum showed their efficient activity and proposed their use as fungicides in crop protection applications.

## 3 Non‐Furanic Biobased Surfactants from HMF

The high reactivity of HMF molecule allows converting it into several building blocks of non‐furanic structures that can be used as starting materials for the synthesis of biobased surfactants. These transformations include ring‐opening of HMF by hydration into levulinic acid, hydrogenative ring‐opening giving 3‐hydroxymetylcyclopentanol, and Diels–Alder reaction of HMF with dienophiles into aromatic compounds. In this section, we discuss different approaches to transform these derivatives into amphiphilic molecules.

### 3.1 Surfactant starting from levulinic acid and esters

Levulinic acid has been classified by the US Department of Energy as one of the twelve promising platform molecules for the synthesis of several chemicals with multiple applications.^[73]^ Acid hydrolysis of HMF leads to the formation of levulinic and formic acids through a double hydration reaction, and when it is performed in presence of alcohols, alkyl levulinates are obtained.^[74]^ Levulinic acid (and its esters) can be converted into biobased amphiphilic molecules through different reactions on the ketone function, such as ketalization, reductive etherification, and reductive amination.

**Scheme 23:**
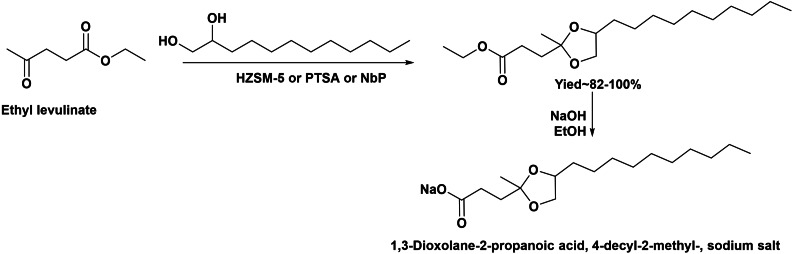
Preparation of anionic green surfactant by ketalization of ethyl levulinate.

For instance, anionic renewable surfactants were prepared by ketalization between ethyl levulinate and long‐chain vicinal diols and subsequent saponification (Scheme 23).^[75]^ Ketalization is an acid‐catalyzed reaction, and ethyl levulinate ester group can undergo competing transesterification, lowering the process selectivity. Then, the acid‐catalyzed ketalization between 1,2‐dodecanediol and ethyl levulinate was carried out in the presence of homogeneous and heterogeneous acid catalysts. After catalyst optimization, niobium phosphate and H‐ZSM‐5 zeolite resulted as the most active and selective catalysts, and high yield of the ketal was achieved after 24 h. After saponification, the 4‐decyl‐2‐methyl‐1,3‐dioxolane‐2‐propanoic acid sodium salt obtained showed a surface tension of 33.5 m Nm^−1^ (1 % in water)_,_ similar to that of the commercial sodium dodecyl sulfate surfactant (35.2 m Nm^−1^). These results showed that the new ketal‐type biobased surfactant prepared from renewable sources constitutes a possible replacement of commercial sodium dodecyl sulfate surfactant. Moreover, the ketal nature of the 4‐decyl‐2‐methyl‐1,3‐dioxolane‐2‐propanoic acid sodium salt converts this amphiphilic molecule into a cleavable surfactant under acidic conditions, which is of great interest from handling and biodegradation standpoints.

**Scheme 24:**
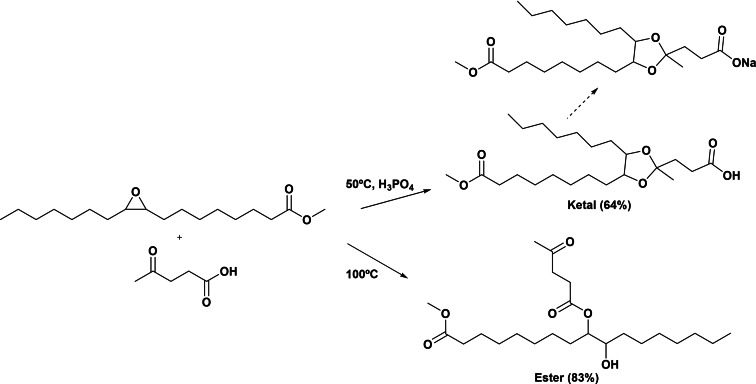
Preparation of new amphiphilic molecules offered new opportunities for the development of new green surfactants from methyl oleate and levulinic acid.

Another different route for the preparation of cleavable amphiphilic molecules involving ketals of levulinic acid was reported by Doll et al.[^76^, ^77^] This strategy involves the acid‐catalyzed ring opening of the methyl oleate epoxide in the presence of levulinic acid. When the reaction was performed in the presence of phosphoric acid as catalyst at 50 °C, the reaction mainly yielded the corresponding ketal (Scheme 24). However, in absence of an acid catalyst and working at 100 °C, the branched ester (83 % of distribution compounds) was the main product. These new amphiphilic molecules offered new opportunities for the development of a new family of green and cleavable surfactants from oleo‐chemicals and sugars.

**Scheme 25:**
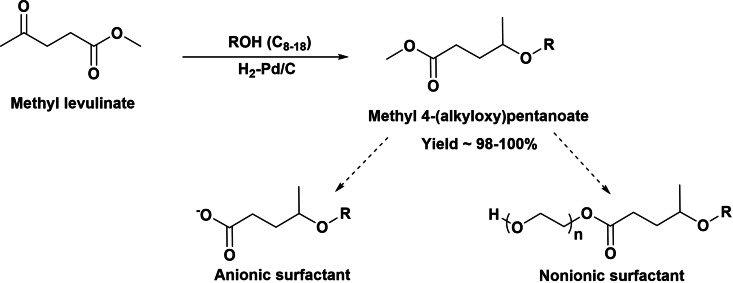
Preparation of methyl 4‐octyloxypentanoate as surfactant precursor by reductive etherification of methyl levulinate with *n*‐octanol.

We have seen that reductive etherification of methyl levulinate with fatty alcohols to produce methyl 4‐alkoxypentanoates has been recently described as a new route for the preparation of precursors of new anionic or nonionic biobased surfactants (Scheme 25).^[78]^ The reaction occurs through the acid‐catalyzed formation of the enol ether intermediate, which is subsequently hydrogenated into the target product. A bifunctional acid/metal catalyst based on Pd nanoparticles supported on carbon was designed to obtain maximum yield and selectivity up to 100 % to the methyl 4‐alkoxypentanoates, while transesterification reactions were avoided. It was shown that Pd species in high‐density planes were the active hydrogenation sites, and an optimum crystal size was found to be approximately 10 nm. The 4‐alkoxypentanoic acid methyl esters can be saponified into 4‐alkoxypentanoic acid alkaline metal salts leading to new green and renewable anionic surfactants. Thus, surface tension of 4‐octyloxypentanoic acid sodium salts (41 m Nm^−1^) determined in aqueous solution (2.5 mm) was lower than that of commercial sodium myristate (59 m Nm^−1^), whereas the CMC was one order of magnitude lower than that of the commercial sample, (5.10^−3^ vs. 2.10^−2^ m). In addition, the methyl 4‐alkoxypentanoates should give access to new family of nonionic surfactants through the transesterification of the methyl ester with polyalcohols or polyoxyethylene glycol. They can be potential substitutes of commercial fossil‐based polyoxyethylene alkyl ethers widely used in industrial and household products and cosmetics formulations.[^79^, ^80^]

**Scheme 26:**
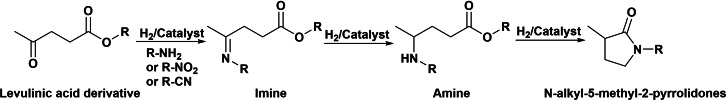
Reductive amination of levulinic acid derivatives to produce *N*‐alkyl‐5‐methyl‐2‐pyrrolidones.

On the other hand, the reductive amination of levulinic acid (and esters) is an important synthesis route for the preparation of *N*‐alkyl‐5‐methyl‐2‐pyrrolidones that constitute important intermediates in the synthesis of pharmaceuticals, agrochemicals, and surfactants.[^81^, ^82^, ^83^, ^84^, ^85^] The proposed reaction pathway for the synthesis of *N*‐substituted‐5‐methyl‐2‐pyrrolidones involves as the first step the acid‐catalyzed formation of an imine intermediate by reaction of a primary amine with the ketone group of the levulinic acid (or ester) followed by hydrogenation of the imine intermediate and subsequent cyclization (Scheme 26).

**Scheme 27:**
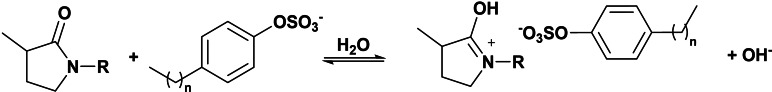
Synergism with linear alkyl‐benzene sulfonate in water.

*N*‐alkyl‐5‐methyl‐2‐pyrrolidones combine the hydrophilic head formed by dipolar pyrrolidone ring and the hydrophobic alkyl group conferring a unique combination of solvency and surface activity. Hence, pyrrolidones have been reported to improve the properties of surfactants such as water solubility, compatibility, and solvency.^[84]^ The *N*‐alkyl chain with approximately C_8_ induces significant surface activity, reaches a maximum for C_12_–C_14_, and then for longer chains drops due to low water solubility. For instance, Surfadone LP‐100 and LP‐300 with C_8_ and C_12_ alkyl chain, respectively, are commercialized by Ashland owing to their great performance due to their high surface activity at low concentration, rapid wetting, synergism with anionic surfactants (as linear alkyl‐benzene sulfonates) to reduce surface tension (Scheme 27), excellent solvency, and biodegradability.[^86^, ^87^, ^88^, ^89^]

Various strategies including the use of H_2_, formic acid, or hydrosilanes as reductants in the presence of a catalyst have been reported, and the latest advances were recently compiled in a Review.^[52]^ However, the use of formic acid or hydrosilanes presents different drawbacks such as corrosiveness, high cost, production of wastes, and harsh reaction conditions. Therefore, gaseous H_2_ is preferred (especially green H_2_) from industrial, economic, and environmental perspectives.

Taking into account the goals of sustainable development, the production of alkyl‐pyrrolidones as nonionic surfactants should be based on efficient and recyclable solid catalysts. Therefore, some relevant examples on reductive amination of levulinic (or esters) with primary amines and H_2_, using robust and recyclable heterogeneous bifunctional catalysts (bearing acid and metal sites, as required by the process) are presented in this section.

For instance, Touchy et al.^[90]^ developed Pt supported heterogeneous and recyclable catalysts for the reductive amination of levulinic acid (Table 3, entry 1). Based on the promoting effect of Lewis acid for the reduction of C=O and C−O bonds,[^91^, ^92^, ^93^, ^94^] the benefits of the presence of transition metal oxides with Lewis acidity (VO_*x*_, CrO_*x*_, MoO_*x*_, WO_*x*_, and ReO_*x*_) over the activity of Pt/TiO_2_ catalysts was explored. Results showed that Pt‐MoO_*x*_ over TiO_2_ was the most active catalyst. This was attributed to the stronger Lewis acidity of Pt‐MoO_*x*_/TiO_2,_ generated by the combination of Pt and Mo metal species, compared with the other transition metal oxide‐based catalysts. Later, Vidal et al.^[82]^ showed that a Pt/TiO_2_ catalyst, where the Pt crystal faces are decorated with TiO_*x*_ species,[^14^, ^95^] resulted a highly active and chemoselective catalyst in the reductive amination of ethyl levulinate with different amines (Table 3, entry 2). The authors demonstrated the formation of protonic acid sites at the interface of Pt/TiO_2_ by hydrogen dissociation on the metal and spillover migration to Lewis acid sites of the support,^[96]^ which promoted the formation of the imine. Further, the same authors reported the one‐pot cascade process for the preparation of *N*‐substituted 5‐methyl‐2‐pyrrolidones starting from ethyl levulinate and nitro compounds in the presence of Pt supported on TiO_2_ nanotubes catalyst (Pt/TiO_2_‐NT). Owing to suitable acidity of the surface catalyst and controlled adsorption of the nitro compounds, the cascade process was successfully performed.^[83]^ More recently, a highly effective TiO_2_ nanosheets‐supported Pt nanoparticles (Pt/P‐TiO_2_) catalyst was reported to prepare *N*‐substituted pyrrolidones by reductive amination of levulinic acid at room temperature and atmospheric H_2_ pressure (Table 3, entries 4 and 5).^[97]^ For instance, the reductive amination of levulinic acid and ester with octylamine can be performed under ambient conditions with high yield (93–97 %) in a few hours. Ammonia temperature‐programmed desorption (NH_3_‐TPD) study showed the stronger acidity of P‐TiO_2_ sample compared with the conventional TiO_2_, which is attributed to its nanosheet structure and high surface area that make a greater number of acidic centers available that should be active for condensation and cyclization reactions.

**Table 3:** Reductive amination of levulinic acid or esters with octylamine with different metal‐based solid catalyst and H_2_ as reductant.^[a]^

Entry	Catalyst [mmol %]	H_2_ [bar]	*T* [°C]	*t* [h]	Substrate^[b]^	*χ* ^[c]^ [%]	Yield [%]	TON^[d]^	Ref.
1	Pt‐MoO_*x*_/TiO_2_ (0.001)	3	100	20	LA	–	95	2150	[90]
2	Pt/TiO_2_ (0.05)	10	120	2	EL	98	98	8540	[82]
3	Pd/ZrO_2_ (0.1)	5	90	12	LA	100	98.7	5625	[98]
4	Pt/P‐TiO_2_ (0.1)	1	RT	3	LA	–	97	–	[97]
5	Pt/P‐TiO_2_ (0.1)	1	RT	10	EL	–	93	–	[97]
6	C‐Au_66_Pd_34_ (0.3)	1	85	12	LA	–	93	–	[99]
7	C‐Au_66_Pd_34_ (0.3)	1	85	12	EL	–	99	–	[99]
8	Cu_15_Pr_3_/Al_2_O_3_ (5)	50	175	20	LA	99.6	94.2	–	[101]

[a] Reaction conditions: solvent‐free. [b] LA=levulinic acid; EL=ethyl levulinate. [c] *χ*=conversion. [d] Turnover number. [a] MeOH as solvent. [b] 1,4‐dioxane as solvent.

Pd‐based catalysts on acidic oxide supports have been also used for reductive amination of levulinic acid to form pyrrolidones with high selectivity under mild reaction conditions. The comparison of the activity of Pd/ZrO_2_, Pd/Al_2_O_3_, and Pd/TiO_2_ catalysts revealed the higher catalytic properties of Pd/ZrO_2_ for amination attributed to higher Lewis acidity (Table 3, entry 3).^[98]^

Recently, the catalytic ability of bimetallic AuPd nanoparticles supported on carbon (C‐AuPd) for reductive amination of ethyl levulinate and levulinic acid reaction was reported (Table 3, entries 6–7).^[99]^ The activity of C‐AuPd was dependent on AuPd alloy composition and was maximized for C‐Au_66_Pd_34_. This was attributed to the existence of a good balance in Pd Lewis acidity and Pd exposure.

From an economic point of view, the development of non‐noble metal‐based catalysts for reductive amination of levulinic acid derivatives is very attractive. Indeed, motivated by sustainable and environmental goals, the development of solid non‐noble metal catalysts based on Earth‐abundant transition metals such as Fe, Ni, or Co has received great attention for reductive amination processes.^[100]^ Recently Cao et al.^[101]^ developed a Cu_15_Pr_3_/Al_2_O_3_ catalyst for the reductive amination of levulinic acid with amines (Table 3, entry 8). Characterization data revealed that the Pr‐doping allowed improving the Cu dispersion and controlled the Cu^0^ particle size. Moreover, the TPD study with ammonia revealed that Pr doping produced a reduction of the amount of weak acid sites and an increase of the amount of strong acid sites. Then, it was concluded that the Cu_15_Pr_3_/Al_2_O_3_ sample exhibited a higher number of stronger acid sites suitable to the adsorption of levulinic acid and amine on the catalyst surface, promoting the condensation and hydrogenation to successfully produce the *N*‐substituted‐pyrrolidone.

The catalytic systems presented above are highly stable and exhibit in general high performance for the synthesis of a variety of levulinic acid‐derived pyrrolidones. However, considering only pyrrolidones useful as non‐ionic surfactants, we have summarized in Table 3 comparative results of these catalytic systems for the synthesis of *N*‐octyl‐5‐methyl‐2‐pyrrolidones.

### 3.2 Preparation of biobased surfactant starting from 3‐(hydroxymethyl)cyclopentan‐1‐ol

**Scheme 28:**
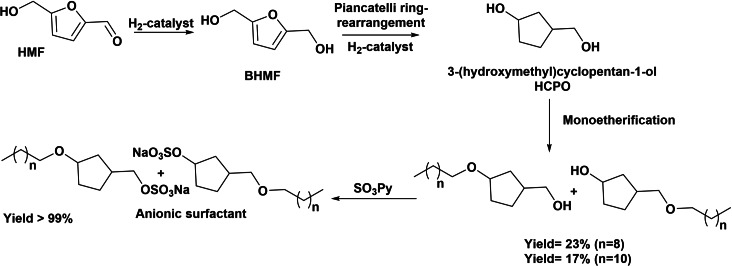
Preparation of alkyl sulfate‐HCPO compounds as new anionic surfactants.

When hydrogenation of HMF is performed in water at high temperature (>140 °C), HMF can be converted into 3‐(hydroxymethyl)cyclopentan‐1‐ol (HCPO). The reaction occurs through the hydrogenation of HMF into BHMF as first step, which subsequently undergoes a Piancatelli ring‐rearrangement into 3‐(hydroxymethyl)cyclopentanone that can be subsequently hydrogenated into HCPO.^[102]^ Recently, preparation of biobased anionic sulfate surfactant by successive monoetherification and sulfatation of HCPO has been reported (Scheme 28).^[42]^ The synthesis of HCPO by ring‐rearrangement of BHMF with 88 % yield was performed in the presence of Pt/SiO_2_+Nd_2_O_3_ catalyst. After Williamson etherification of HCPO, monoethers (17–23 % yield) with a primary/secondary ratio of 70 : 30 were obtained. Subsequent sulfatation of the free hydroxy group led to new amphiphilic molecules. It was showed that dodecyl sulfate‐HCPO derivative was not soluble in water, while the evaluation of CMC and interfacial tension towards isopropyl myristate showed that the decyl sulfate‐HCPO derivative exhibited relatively high CMC (≈0.77 g L^−1^) and interfacial tension (6.1 m Nm^−1^), which were considerably higher than commercial LAS (C_10–14_; CMC≈0.08 g L^−1^ and surface tension≈0.9 m Nm^−1^) and fatty alkyl ether sulfates (FAES, C_12–14_; CMC close to 0.05 g L^−1^ and surface tension close to 3.9 m Nm^−1^).

## 4 Aromatic Biobased Surfactant Precursors

**Scheme 29:**
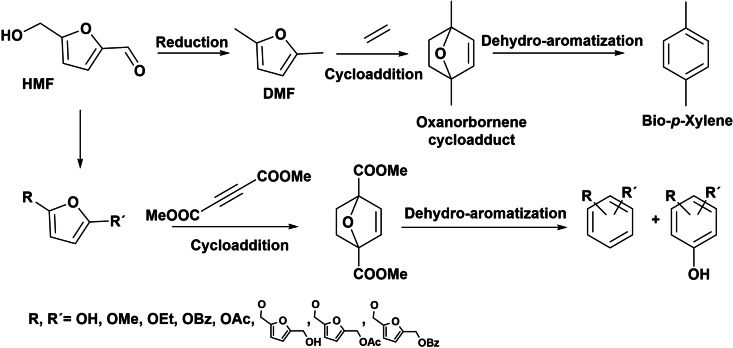
Production of aromatic compounds from aromatic compounds from biobased furans derived from HMF.

Alkyl benzene sulfonates are an important class of anionic surfactants, in which the aromatic moiety is sourced from fossil fuels. However, due to the depletion of fossil resources, in the last years research has been focused on the production of aromatics from biomass. Multiple strategies have been explored for the direct replacement of aromatic compounds from biomass by thermochemical (pyrolysis, gasification), biological, and catalytic processes.[^104^, ^105^, ^106^, ^107^] However, selectivity to single aromatic compounds remains a challenge due to complex mixtures obtained from thermochemical and catalytic processes. Therefore, different approaches have been followed to produce aromatic compounds from biobased furans as such HMF and to pave the path to a renewable chemical industry and lower carbon footprint.[^107^, ^108^] Furanics‐to‐aromatics conversion can be achieved by the Diels–Alder cycloaddition of furans (derived from HMF) with dienophiles and subsequent aromatization (Scheme 29).[^107^, ^109^] Particularly, acid zeolites bearing Brønsted and Lewis acid sites have shown high potential as catalysts for the furan aromatization process. For instance, biobased *p*‐xylene has been produced by Diels–Alder addition of bio‐ethylene to 2,5‐dimethylfuran (DMF) in the gas phase, at 250–300 °C and high ethylene pressure (up to 62 bar) in the presence of HY or H‐Beta zeolites, achieving 60 and 90 % selectivity to *p*‐xylene, respectively, at >95 % conversion (Table 4, entries 1 and 2).[^110^, ^111^] More recently, a combination of theoretical and experimental results was used to design and synthesize an efficient ab‐initio^[112]^ zeolite catalyst for Diels–Alder reaction.^[113]^ According to experimental and computational studies, the limiting step of the overall process is the non‐catalyzed Diels–Alder cycloaddition, where the transition state and the final cycloaddition adduct have similar stability. Then, the zeolite design was based on the concept of using organic structure‐directing agents that mimic the transition state, proposing a molecule derived from 1,4‐diazabicyclo[2.2.2]octane (DABCO) that could mimic the oxanorbornene cycloadduct intermediate (Scheme 29). Thus, the study revealed that a directly synthesized ITQ‐2 zeolite resulted an efficient catalyst for this reaction, giving enhanced reaction rates in comparison to other zeolite structures (Beta and FAU; Table 4, entries 3–6). Also, Lewis acid zeolites such as Zr‐Beta were very active and selective in the Diels–Alder cycloaddition of DMF and ethylene, achieving 90 % *p*‐xylene (at 99 % DMF conversion) under similar reaction conditions (Table 4, entry 7).^[114]^ In the same way, in the presence of P‐containing zeolite Beta, 97 % *p*‐xylene yield was achieved (Table 4, entry 8).^[115]^ Recently, the production of *p*‐xylene with 92 % yield by Diels–Alder reaction with renewable DMF and acrylic acid in the presence of heterogeneous Bi‐BTC catalyst (metal‐organic framework) under mild reaction conditions (160 °C, 10 bar) has been reported (Table 4, entry 9).^[116]^ The low activation energy calculated in the presence of Bi‐BTC catalyst allowed producing valuable BTX (benzene/toluene/xylene) compounds under mild conditions.

**Table 4:** Diels–Alder cycloaddition of DFF (derived from HMF) with dienophiles and subsequent aromatization.

Entry	Catalyst	Dienophile	*P* [bar]	*T* [°C]	*t* [h]	Solvent	*χ* [%]	Yield [%]	Sel. [%]	Ref.
1	HY	ethylene	62	250	24	heptane	95	≈57	60	[111]
2	HB	ethylene	62	250	24	heptane	99	90	90	[110]
3	DS‐ITQ‐2	ethylene	52	240	3	heptane	35	≈16	46	[113]
4	MCM‐22	ethylene	52	240	3	heptane	17	≈6	35	[113]
5	USY	ethylene	52	240	3	heptane	17	≈8	47	[113]
6	HB	ethylene	52	240	3	heptane	26	≈13	49	[113]
7	Zr‐Beta	ethylene	62	250	24	heptane	83	61	74	[114]
8	P‐Beta	ethylene	62	250	20	heptane	99	97	98	[115]
9	Bi‐BTC	acrylic acid	10	160	24	acetone	99	92	93	[116]

**Scheme 30:**
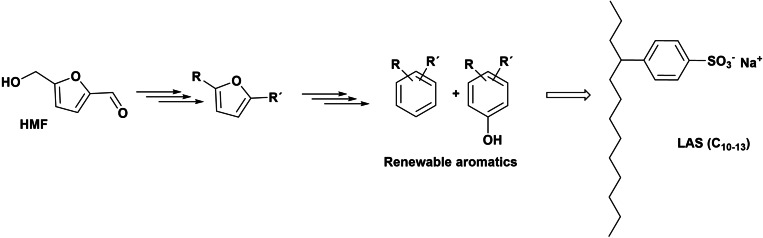
HMF as precursors of LAS.

These examples show that the production of biobased aromatics from HMF derivatives can be a feasible alternative to fossil fuels for the preparation of LAS analogues (Scheme 30).

## 5 Summary and Perspectives

As mentioned in this Review, surfactants find applications in our daily life and in numerous industry sectors, with the global surfactant market size growing steadily. In order to achieve sustainable development, the chemical industry is constantly working on research and development of biobased alternatives to petroleum‐based chemical platforms. Consequently, the interest in producing and using 5‐hydroxymethylfurfural (HMF) as a renewable platform chemical for the production of a wide range of chemicals and materials has grown considerably in recent decades. Therefore, in view of the high demand for surfactants and sustainable development goals, there has been a continuous interest in the last twenty years to design and synthesize new biobased surfactants to limit their environmental impact. In this Review, we have shown the important role of HMF as platform molecule in the synthesis of bio‐based surfactants, not only from the perspective of renewability and carbon footprint, but also by improving biodegradability. The successful synthesis of biobased anionic, nonionic, cationic, and amphoteric oleofuran and tetrahydrofuran surfactants starting from HMF with suitable surfactant properties that cover a wide range of applications has been demonstrated. In the same way, the preparation of advanced amphiphilic molecules, such as gemini surfactants with enhanced and unusual features, or cleavable surfactants with improved biodegradability and elimination, has been thoroughly described. Starting from other HMF derivatives such as levulinic acid (or esters), 3‐(hydroxymethyl)cyclopentan‐1‐ol, or biobased aromatics, new amphiphiles can also be constructed covering different class of surfactants. Nevertheless, many efforts are still needed to improve the synthesis routes avoiding stoichiometric reactions in favor of catalytic routes. In this sense, the design of suitable heterogeneous catalysts and the development of new one‐pot cascade processes (chemo and chemoenzymatic processes) will allow to accomplish efficient, sustainable, and environmentally friendly strategies. Furthermore, since surfactants are major pollutants in the environment, the inclusion of sensitive or labile fragments in the molecule backbone that allow to neutralize (loss of surface tension activity) and/or degrade them quickly should be considered for designing new surfactants molecules.[^117^, ^118^] In this sense, surfactants degradation by varying chemical conditions such as pH or redox[^119^, ^120^] conditions, ionic environment (salinity), light,^[121]^ temperature, or ozone are alternative methods to biodegradation. Among these, natural light provides a great advantage for a harmless degradation of surfactants without alteration of the environment. For example, the incorporation of photoactive or photolabile component such as diazosulfonates in the surfactant is key to cleave them under UV irradiation.[^121^, ^122^, ^123^] Heterogeneous photocatalysis has also been pointed out as an effective method for surfactant neutralization in high concentration, such as the decomposition of the aromatic moiety of anionic and cationic surfactants in the presence of TiO_2_ and solar exposure,^[124]^ the photocatalytic degradation of non‐ionic aromatic surfactants using ZnO,^[125]^ and the degradation of dodecyl benzene sulfonate (laundry wastewater) under UV/TiO_2_/H_2_O_2_ and UV/Fe^2+^/H_2_O_2_ processes.^[126]^ Thus, photocatalysis and incorporation of photosensitive moieties should constitute important approaches in the development and life cycle of new amphiphiles.

**Scheme 31:**
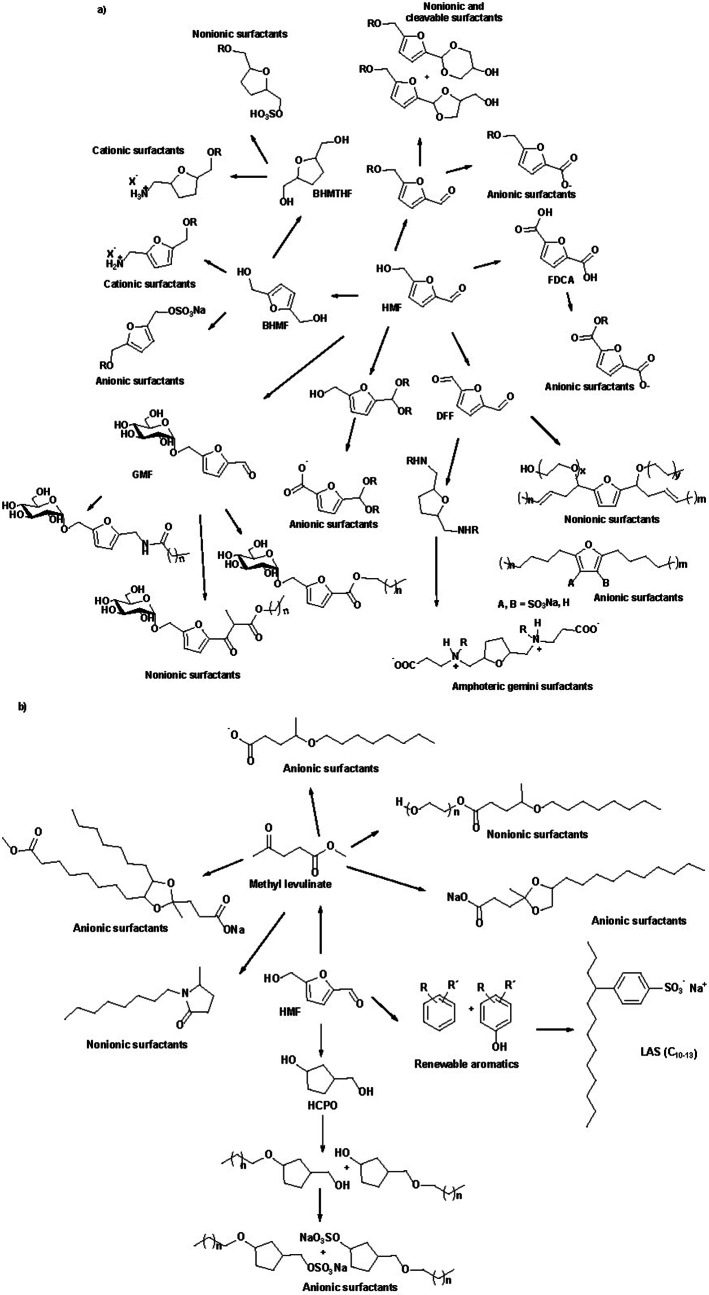
New anionic, cationic, and non‐ionic biobased surfactants prepared starting from (a) HMF or (b) non‐furanic derivatives.

The different routes taken to convert HMF into valuable surfactants are summarized in Scheme 31.

## Conflict of interest

The authors declare no conflict of interest.

## Biographical Information

*Avelino Corma, Professor and founder of the Instituto de Tecnología Química (CSIC‐UPV) in Valencia (Spain), has carried out research in heterogeneous catalysis in academia and in collaboration with companies. He has worked on fundamental aspects of acid‐base and redox catalysis with the aim of understanding the nature of the active sites and reaction mechanisms. With these bases has developed catalysts that are being used commercially in several industrial processes. He is an internationally recognized expert in solid acid and bifunctional catalysts for oil refining, petrochemistry, and chemical processes. He has published more than 1000 research papers and is inventor on more than 130 patents*.

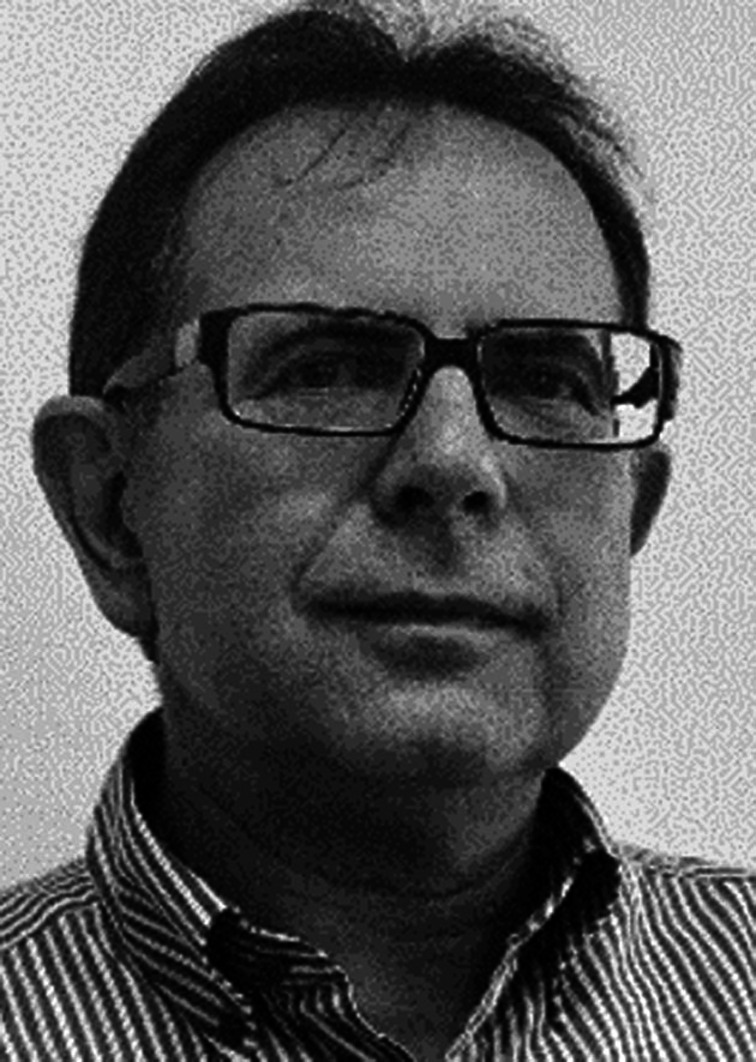

## Biographical Information

*Sara Iborra is Full Professor at the Chemistry Department of the Technical University of Valencia. She received her Ph.D. in 1987 at the Universidad de Valencia, and in the same year, she joined to the Chemistry Department of the Technical University of Valencia as Assistant professor. She is member of the Institute of Chemical Technology (ITQ) at the Technical University of Valencia since 1991, where she works in the research group of Prof. Avelino Corma. The main focus of her current research involves the application of heterogeneous catalysts (acid‐base and redox) to the synthesis of fine chemicals and biomass transformation*.

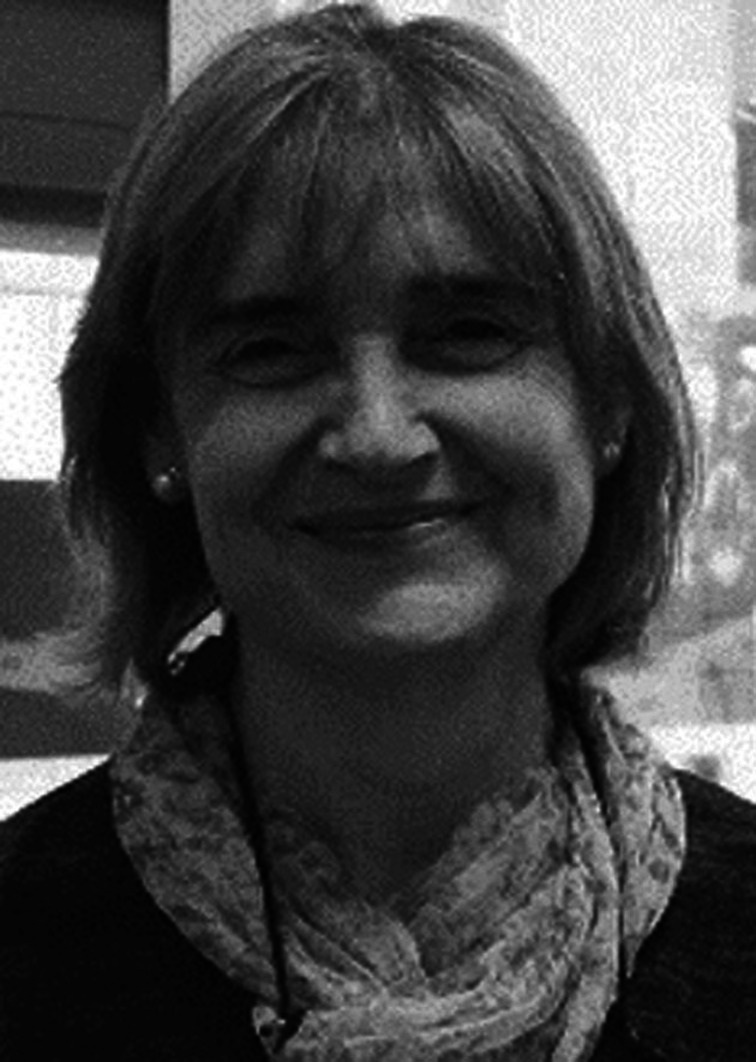

## Biographical Information

*Alexandra Velty graduated in Chemical Engineering at the University of Rennes and received her B.Sc. in Chemistry at the University of Nancy (France). She completed her Ph.D. under the supervision of Prof. Avelino Corma at the Instituto de Tecnología Química (ITQ, UPV‐CSIC) in Valencia (Spain) in 2003 in the field of heterogeneous catalysis and green chemistry for the preparation of fine chemicals. Until now, she is working in Prof. Avelino Corma's group as a permanent member of the Polytechnic University of Valencia. Her research interests focus on heterogeneous catalysis and sustainable chemistry for biomass transformation and fine chemicals preparation*.